# Determining Immunoglobulin Content of Bovine Colostrum and Factors Affecting the Outcome: A Review

**DOI:** 10.3390/ani11123587

**Published:** 2021-12-18

**Authors:** Johanna Ahmann, Julia Steinhoff-Wagner, Wolfgang Büscher

**Affiliations:** 1 Institute of Agricultural Engineering, University of Bonn, 53115 Bonn, Germany; buescher@uni-bonn.de; 2 Institute of Animal Science, University of Bonn, 53115 Bonn, Germany; jste@itw.uni-bonn.de

**Keywords:** colostrum quality, refractometer, colostrometer, calf husbandry, radial immunodiffusion, colostrum management

## Abstract

**Simple Summary:**

Colostrum management plays an essential role in calf husbandry and strongly influences the calf. The immunoglobulin concentration denominates the quality of the colostrum, which is influenced by numerous factors. Therefore, the measurement of the immunoglobulin concentration is important. This review provides an overview of measurement methods for estimating the immunoglobulin concentration in bovine colostrum. In addition, influencing factors are identified and their impact on the immunoglobulin concentration is discussed. Radial immunodiffusion and the Enzyme-linked immunosorbent assay are the most commonly used direct measurement methods. A refractometer and a colostrometer are practical indirect on-farm instruments that can be used to estimate the immunoglobulin concentration. External characteristics such as viscosity or color allow for an initial assessment but are too inaccurate. Animal-related factors such as colostrum yield, parity, and breed influence the immunoglobulin concentration. In addition, environmental factors are also important. The duration between birth and first feeding postpartum is important for the supply of colostrum with a sufficient immunoglobulin concentration. The influence of treatment methods such as freezing and thawing, on the other hand, depends strongly on the procedure and does not necessarily lead to a reduction in the immunoglobulin concentration. The influencing factors are complex and newer ones, such as genetics, have not yet been sufficiently investigated.

**Abstract:**

The immunoglobulin concentration in bovine colostrum should be measured to ensure feeding with sufficient immunoglobulins (≥50 mg immunoglobulin G mL^−1^). Adequate feeding prevents diseases, promotes development, and has a positive influence on the adult animal. Indirect and direct measurement methods are available for this purpose. Direct measurement methods cannot be easily used in practice; therefore, farmers use indirect methods such as a colostrometer and a refractometer. Many factors influence the immunoglobulin concentration of colostrum; some of them have already been intensively researched. In particular, lactation and temporal aspects play an essential role. Newer aspects such as dry period, seasonal influences, and genetics are gaining importance, but their impact on immunoglobulin content has not been sufficiently investigated. Developments are still needed, especially in data management. This review analyzes the outcome of different studies on the indirect and direct measurement methods and discusses different factors influencing the immunoglobulin concentration of bovine colostrum.

## 1. Introduction

An adequate and timely supply of colostrum, within the first hours after birth, is essential for newborn calves and their later development [1,2]. The bovine placenta prevents the transfer of immunoglobulins (Ig) between the mother cow and her fetus. The placenta membranes have limited permeability, such that only gases and small molecules are able to pass through the membranes. Ig cannot pass through the membranes and because of that, calves are born with a minimal antibody level. However, the rapid intake of colostrum, which contains an adequate level of Ig, provides the calf with passive immunity [2].

The most important Ig in cow colostrum are immunoglobulin G (IgG) (with the subtypes IgG_1_ and IgG_2_), immunoglobulin A (IgA), and immunoglobulin M (IgM). IgG is the main component of cattle colostrum, accounting for 85 to 95% of the total Ig concentration. In colostrum, IgG_1_ dominates, whereas the level of IgG_2_ is much lower. IgM is the second most common Ig, followed by IgA [3,4,5,6]. The colostrum quality is an important factor in colostrum management, whereby the Ig concentration determines the quality of the colostrum. Generally, “good” colostrum has an IgG concentration ≥50 mg mL^−1^ [7,8]. Since the central part of Ig is IgG, the IgG concentration is often measured, rather than the total Ig concentration. A higher IgG_1_ concentration in colostrum leads to a higher concentration of IgG_1_ in the serum of calves [9]. However, the Ig concentration in colostrum can vary greatly from cow to cow, with various factors influencing the concentration. Thus, different studies have determined widely varying concentrations of Ig in the colostrum of cows [10,11]. Table 1 lists the quantity and ratio of Ig measured across different studies.

To prevent negative consequences, calves should consume colostrum as soon as possible after birth, particularly since the Ig concentration in the colostrum decreases significantly with each hour after birth [5,14]. Additionally, the permeability of the calf’s intestinal mucosa for Ig molecules decreases rapidly after 12 h, and usually disappears entirely on the second day of life [2]. Therefore, the timely feeding of colostrum ensures an adequate uptake of IgG_1_ via the colostrum. An additional critical factor is the quantity of colostrum that the calf consumes during their first feeding after birth. Farmers should feed calves 10 to 12% of their body weight in colostrum in the first feeding to achieve a sufficient uptake [15]. The calves that consume 4 L of colostrum have a higher serum Ig concentration than the calves that consume 2 L of colostrum [16]. Moreover, calves supplied with colostrum containing a sufficient Ig concentration immediately after birth are less susceptible to diarrhea and lung diseases; these calves also develop better and show stronger growth [2]. Good colostrum management also leads to reduced morbidity and mortality in the first week of life [17]. In addition, the supply of colostrum influences further rearing; an inadequate supply leads to a later first calving age, as the required body weight is reached later [18]. The supply of colostrum also influences the adult animal. Cows that received an additional 2 L of colostrum as calves produced 1349 kg more milk in the second lactation, compared to animals that received lower quantities of colostrum (9516 ± 251 vs. 7526 ± 252 kg, *p* < 0.05); however, the difference is smaller during the first lactation, although the calves who received 2 L still produced more milk (7848 ± 253 vs. 7526 ± 252 kg) [19]. In addition, the veterinary costs for calves that consume a greater volume of colostrum are lower than for calves that receive only 2 L. Calves with a lower colostrum intake require repeated treatments and monitoring to treat diseases, leading to increased veterinary costs [19].

An insufficient amount of IgG in the calf’s blood 24 to 48 h after birth is referred to as a “Failure of Passive Transfer” (FPT) [20]. An IgG value < 10 mg dL^−1^ in the blood serum is often cited as an indicator of an FPT [21,22,23,24]; this FPT cut-off point is widely used to assess antibody uptake. An FPT increases economic losses. An insufficient supply of colostrum results in 60 to 80 € of extra costs per dairy or beef calf. If the prevalence of an FPT is high, these costs can rise to 95 € per dairy calf or 132 € per beef calf [25]. Nonetheless, a high Ig concentration in the colostrum does not automatically lead to a high Ig concentration in the calf’s serum; different factors also influence the absorption of Ig [26,27], but these will not be discussed in this review.

For the reasons outlined above, controlling both the Ig concentration in the colostrum and colostrum intake is of great importance to calf rearing. The Ig concentration of the colostrum could be easily measured after milking. Nevertheless, only a few farms are undertaking this determination [2,3,26]. There are various direct and indirect measurement methods to estimate the concentration of Ig in colostrum. However, there is currently no direct measurement method that can be applied on farms; all the on-farm tools belong to the indirect measurement methods [27].

This article reviews the indirect and direct measurement methods to define the Ig concentration in colostrum. It compares the techniques in terms of their application under practical conditions and derives possible uses and development needs. In addition, the positive and negative aspects of the direct and indirect measurement methods, and the factors influencing the Ig concentration in colostrum, are discussed.

## 2. Methods for Measuring the Immunoglobulin Concentration of Colostrum

There are direct and indirect measurement methods to determine the Ig concentration in colostrum. Direct methods measure the Ig concentration, whereas indirect methods allow conclusions about the Ig concentration based on correlated properties. The indirect methods, for instance, are based on the change in the physical and chemical properties of colostrum as a liquid, whose specific gravity, density, or viscosity changes depending on the Ig concentration [2,3]. On-farm tools, whether direct or indirect, should be easy to use, effective, and accurate. In addition, results should be available quickly and the costs should be kept to a minimum [10,20]. We chose the following order in this review based on the frequency of the measurement methods in the literature used.

### 2.1. Direct Measurement Methods

#### 2.1.1. RID and ELISA

Radial immunodiffusion (RID) is considered the gold standard for determining the Ig concentration in colostrum [10,22,28]. RID is an immunoprecipitation method for the quantitative determination of antigens in a sample. The antigen-containing samples (e.g., colostrum) are pipetted into the round punched holes of an antibody-containing agarose gel plate. The antigens diffuse circularly into the gel. This produces precipitate rings whose diameter (raised to the square) is proportional to the amount of antigen in the sample [29].

The Enzyme-linked immunosorbent assay (ELISA) is frequently used to quantify Ig in colostrum [30,31,32]. An ELISA is based on the antigen-antibody reaction and is a method for detecting and quantifying Ig. Immune complexes are formed, which additionally combine with enzymes. Based on this binding, the immune complexes can then be measured [29].

In a study by Gelsinger et al. (2015) [33], the IgG concentration in colostrum was measured with RID and an ELISA. Due to the high number of retests when the samples were analyzed using ELISA in this study, RID was considered the more consistent method. In addition, heating the colostrum resulted in a lower IgG concentration as measured using ELISA, whereas it did not induce any changes when measured using RID. However, there was a higher correlation between the values measured before and after heating using ELISA, compared to those measured using RID. The authors described this result as surprising, as the values measured by RID did not change after heating. In their view, this illustrates that the effect of heating on the protein composition in colostrum has a different impact on RID and ELISA [33].

Zobel et al. (2020) [34] also measured the IgG concentration in colostrum and found a lower test performance using ELISA, compared to RID. The ELISA results were also not repeatable using the RID method. These results are similar to the previously discussed research, despite using a different animal species (caprine). Based on their results, the authors do not recommend a direct comparison between the caprine IgG concentration recorded in different studies using different measurement methods (e.g., ELISA vs. RID) [34]. These findings differ from the work of Dunn et al. (2018) [35], in which the researchers assumed that the differences in correlations between different studies were also due to the specific kits used in each study. On average, the IgG concentrations measured using an ELISA were 1.8 times lower than those obtained using RID. A wide level of agreement (12.61–51.17) between RID and the ELISA was found in terms of the IgG concentration in colostrum. The authors suggested that this variation is due to the different dilutions of the samples. The samples were more diluted for the ELISA than for the RID samples [35]. Although the authors did not address sensitivity, it may be an additional reason for differences across the results of the two methods. Table 2 shows the different correlations between the RID and ELISA results that have been calculated in different studies. Based on these trends, the quality of the Ig may matter if there is no relationship.

As mentioned above, in the literature, colostrum with a minimal IgG value ≥ 50 mg mL^−1^ is considered to be of good quality. This cut-off value is largely quoted for measurements with RID as a standard. As such, new limits may need to be set for the ELISA as a standard method [33]. None of the authors who used the ELISA experienced minimum values for the ELISA.

Even though RID and the ELISA are both very time-consuming, they are very sensitive laboratory methods [29]. Assuming calves should consume colostrum with a sufficient Ig concentration no later than 3 h after birth, these methods are unsuitable for rapid, practical use [1,23,33,36]. In addition, specific reagents with a limited shelf life and specific equipment are required to perform both procedures; therefore, the user must also be skilled in handling these materials; this is hardly feasible in practice [23,33,36,37]. Furthermore, the immunoprecipitated rings in simple RID are not stable, as the antibody concentration in the gel is constant. Therefore, evaluation must be performed at the exact correct time [29].

Large amounts of reagent-antibodies are required for RID; therefore, the cost of RID is relatively high [29]. The prices of the different assays vary greatly, ranging from 2.00 to 13.65 $ per test. For RID, the costs depend on the number of samples simultaneously tested since the standards must be determined each time [36]. For an ELISA test, prices also vary widely; according to German trade prices, the cost varies from 4 to 7 € per test. In the future, the ELISA could be an economical alternative to RID [33], since many samples can be analyzed simultaneously, and the process can be almost entirely automated [38]. However, if only individual calvings are considered, it is questionable whether this is necessary or not.

Furthermore, the correlations in measurements across the studies differ greatly and do not show uniformity. These differences do not permit the formulation of any conclusions about whether the ELISA is suitable for adequately determining the Ig concentration in colostrum. Nevertheless, in research studies, ELISAs are commonly used to determine Ig concentration and could equally be considered the gold standard. A relatively high correlation was achieved in one study, in which the authors repeatedly referred to the influence of the specific test kits. Therefore, testing ELISA kits prior to their use should be considered.

#### 2.1.2. Turbidimetric Immunoassay

Another method for determining the Ig concentration in colostrum is the turbidimetric immunoassay (TIA). A TIA is based on an antigen–antibody reaction and the resulting immune complexes that can absorb and scatter light. The light absorption is measured photometrically and is proportional to the antigen concentration over a wide range. More precisely, the increase in light attenuation (extinction) per minute is measured. Photometers and photometric analyzers can be used to perform a TIA [29].

Quigley et al. (2013) [8] additionally tested a TIA for measuring the IgG concentration in colostrum and were able to demonstrate a high correlation (r = 0.87; *p* < 0.01) between the IgG concentration in the colostrum determined by RID and the TIA. However, the TIA underestimated the IgG concentration compared to RID. Among the samples measured using the TIA, significantly more were below the limit of 50 mg mL^−1^ compared to those measured using RID [8]. Alley et al. (2012) [39] also calculated a very strong correlation between RID and the TIA (r = 0.99; *p* < 0.05) for measuring IgG concentration in colostrum [39].

In a comparison between a TIA and an ELISA, the IgG concentrations measured using the TIA (49.8 ± 26.3 mg mL^−1^) were, on average, 21 mg mL^−1^ lower than the values measured using the ELISA (70.8 ± 27.7 mg mL^−1^). It was not only the difference that proved to be statistically significant (*p* < 0.0001) but also the correlation between these two methods (r = 0.74; *p* < 0.001). There were clear differences in the direct comparison of the measured values despite the correlations, especially for samples with a high IgG concentration in the ELISA. The sensitivity of the TIA was 1.0 and the specificity 0.40. Sensitivity described the proportion of the TIA test results that indicated an inadequate (≤50 mg IgG mL^−1^) colostral IgG concentration and was confirmed as such by the ELISA. The specificity described the proportion of test results using the TIA that indicate an adequate (>50 mg IgG mL^−1^) colostral IgG concentration and was confirmed as such by the ELISA [40]. In a study by Quigley et al. (2013) [8], the values determined using the TIA were lower than RID. Alley et al. (2012) [39] concluded that a TIA does not show better correlations with the direct measurement method ELISA than with established, less expensive, and indirect methods (e.g., colostrometer). Moreover, this is also a laboratory method that, similar to RID and the ELISA, cannot be used in practice without certain preconditions. The results of the TIA may also be affected by the non-IgG components. Quigley et al. (2013) [8] stated that components such as fat could affect the turbidity and thus, the result of a TIA. Furthermore, the cost-effectiveness of this method is questionable.

#### 2.1.3. Infrared Spectroscopy

Infrared (IR) spectroscopy is based on transitions between vibrational levels. Molecules can carry out such vibrational transitions; however, two things must be fulfilled for this. Firstly, the light of a suitable wavelength must be irradiated. Secondly, a change in the dipole moment must be associated with the oscillation of the molecules. The latter condition is called IR activity. The spectral range that connects to visible light toward longer wavelengths (approximately 760–800 nm) is called “infrared”. This range is divided into the near IR (NIR, 760–3000 nm), the mid-IR (MIR, approximately 3–30 µm), and the far IR (FIR, approximately 30–1000 µm). The division into these three ranges is made because different forms of vibration of the molecules occur in the different ranges [41].

Elsohaby et al. (2018) [42] observed a correlation of r = 0.88 between the measured IgG concentration of RID and IR in fresh colostrum. For heated colostrum, the correlations varied between r = 0.85 and r = 0.70, depending on the period and temperature. The lowest correlation was found at 63 °C/60 min (r = 0.70). For IR and fresh colostrum, the sensitivity and specificity were 0.82 and 1.00, respectively, (cut-off point of 50 mg IgG mL^−1^) and the accuracy was 0.92. All three values are affected by heating; therefore, the sensitivity, specificity, and accuracy at 63 °C/60 min were 0.63, 0.83, and 0.80, respectively. Heating colostrum at 60 °C for 30 or 60 min does not seem to affect the IR results. However, when they raised the temperature to 63 °C for the same amount of time, inaccuracies in the IgG concentration (measured using IR) were observed. Another study investigated the potential of transmission infrared (TIR) spectroscopy to determine IgG concentration in colostrum from dairy and beef cows. A total of 430 samples were analyzed and RID was the comparative method. The correlation measured with RID and TIR between the IgG concentration of two different colostrum sets was 0.84 and 0.96, respectively [43]. The correlations between RID and TIR concerning the IgG concentration are, in part, higher than the correlations measured between RID and the colostrometer [5,7,31,44] or the refractometer [11,20,34,45]. Another study determined good agreement between the different statistical parameters calculated for RID and IR. The correlation between these two methods was 0.91.

Furthermore, a sensitivity of 0.90, a specificity of 0.92 and an accuracy of 0.90 were calculated if IR is used regularly. Using a cut-off value of <50 mg IgG mL^−1,^ IR classified eight colostrum samples as false positives and 16 samples as false negatives (*n* = 250) [46]. IR spectroscopy cannot be used in practice without further prerequisites, as this is also a laboratory method. However, the method appears to be more accurate than indirect measuring instruments, and the results are promising for future studies.

Nevertheless, performing IR requires expensive equipment. A spectrometer with which the IR spectra are acquired can cost up to 2000 € depending on the equipment. These are certainly laboratory devices that will not find purchase applications on farms.

### 2.2. Indirect Measurement Methods

#### 2.2.1. Refractometer

An indirect tool for the measurement of the Ig concentration in colostrum is the refractometer, which measures the concentration of dissolved substances in liquids. A refractometer can be used to determine the refractive index, permitting conclusions about the density of the liquid to be made. The concentration of the ingredients (e.g., Ig) influences the density of the liquid (e.g., colostrum). As such, by measuring the density, conclusions can be drawn about the concentration of the ingredients [8,47]. The density of a liquid depends on its temperature, with density decreasing with an increasing temperature [48]. Therefore, the temperature of the colostrum can influence the result. Most refractometers include automatic compensation for the temperature [49]. Refractometers provide results expressed as %Brix, wherein the Brix value corresponds to the proportion of dry matter percentage [8,47].

A differentiation is made between optical and digital refractometers. Optical refractometers must be held in the direction of a light source, then the measured value can be read in %Brix. Digital refractometers automatically display the result in digital form. Before the Ig concentration in colostrum was measured, refractometers were mainly used to measure the Ig levels in blood serum [50,51]. Refractometers can, therefore, also be used to examine the possibility of an FPT [52].

Some studies have investigated the suitability of both optical and digital refractometers for determining the IgG concentration in colostrum [11,20,34], whereas others have only used one type of refractometer [7,8,42,45]. In studies analyzing the ability of refractometers to determine the Ig concentration in colostrum, the sensitivity ranged from 0.56 to 1.0 for optical refractometers and from 0.66 to 1.0 for digital refractometers. Optical refractometers had specificities of 0.63 to 0.90, whereas digital refractometers had specificities of 0.65 to 0.83. Sensitivities varied more in optical refractometers than digital refractometers. For both types of refractometers, the ranges in values for specificity and sensitivity were wide. Table 3 outlines the different sensitivities, specificities, and correlations from the studies included in this review.

Most studies have demonstrated correlations of around 0.7 between measurements obtained via RID and both optical and digital refractometers [11,20,34,45]. Elsohaby et al. (2017) [11] and Bielmann et al. (2010) [20] determined higher correlations between the concentrations determined via RID and a digital refractometer than RID and an optical refractometer. On the one hand, Zobel et al. (2020) [34] determined a higher correlation for the optical refractometer (r = 0.73) and RID than the digital refractometer. This value was confirmed by Elsohaby et al. (2018) [42] (*n* = 60), whereas Quigley et al. (2013) [8] calculated a slightly higher correlation (r = 0.75; *n* = 183). On the other hand, Bartier et al. (2015) [7] showed a lower correlation (r = 0.64) between the IgG concentrations detected using RID and a digital refractometer (*n* = 460). Using the ELISA as a standard, Lemberskiy-Kuzin et al. (2019) [31] validated an optical refractometer; they found R^2^ values of 0.43. On the other hand, in studies by Bielmann et al. (2010) [20] and Zobel et al. (2020) [34], R^2^ values of 0.56 and 0.53, respectively, were calculated.

Bielmann et al. (2010) [20] and Zobel et al. (2020) [34] found high correlations between the Ig concentrations determined via the two refractometer types. Bielmann et al. (2010) [20] noted a correlation of 0.98 (*p* < 0.001) for fresh colostrum, and 0.97 (*p* < 0.001) for frozen colostrum. For fresh colostrum, Zobel et al. (2020) [34] found a similar correlation between measurements taken via a digital and optical refractometer (r = 0.99). Additionally, Bartens et al. (2016) [10] investigated the intra-observer reliability of both types of refractometers. The intraclass correlation coefficients were 0.97 (confidence interval (CI) = 0.95–0.98) and 0.98 (CI = 0.97–0.99) for the optical and digital refractometer, respectively.

Elsohaby et al. (2018) [42] noted stronger correlations between the IgG concentration measured using RID and an optical and digital refractometer for unheated colostrum (r = 0.73 and 0.74) than for colostrum heated to 63 °C for 30 or 60 min.

Based on their study, Bielmann et al. (2010) [20] established an optimal threshold of 22% Brix to detect colostrum with an IgG concentration ≥50 mg Ig mL^−1^. Further studies have provided different cut-off points for refractometers. For instance, Bartens et al. (2016) [10] calculated an optimized cut-off point of 27% Brix for an optical refractometer; this value is similar to that proposed by Dunn et al. (2018) (27.3% Brix) [35]. Bartens et al. (2016) [10] obtained a cut-off point of 23.4% Brix for a digital refractometer; comparable values were found in studies by Bartier et al. (2015) [7] (23% Brix), Bielmann et al. (2010) [20] (22% Brix), Chigerwe et al. (2008) [1] (22% Brix), and Elsohaby et al. (2017) [11] (24% Brix). Nevertheless, other studies also describe lower cut-off points, such as 20.6 and 21.9% Brix [5,45]. Across all studies, the most commonly used cut-off point is ≥22% Brix; however, a meta-analysis of the accuracy of refractometers in detecting colostrum with an IgG concentration ≥50 mg mL^−1^ demonstrated that a cut-off point of 22% Brix leads to a significant number of false-negative samples [55]. This prevalence of false negatives seems to be particularly high when the prevalence of good colostrum is high. As a result, a cut-off point of 22% Brix can lead to poor colostrum ratings, even when the sample contains a sufficient IgG level. Buczinski et al. (2016) [55] consider a Brix value < 18% Brix useful for filtering out colostrum with an insufficient Ig concentration, whereas colostrum in the range of 18–22% Brix should be considered suspect, and colostrum with a Brix value ≥ 22% Brix should be used.

Rayburn et al. (2019) [53] used a digital refractometer to examine the IgG concentration in colostrum and transition milk up to the fifth milking. For the first milking, a cut-off point of 19.3% Brix was used to detect colostrum with IgG levels of at least 50 mg mL^−1^; a sensitivity of 0.83 and specificity of 0.51 were obtained. For the second milking, a cut-off point of 14% Brix was chosen as a value for 25 mg IgG mL^−1^ milk, whereas a cut-off point of 12.3% Brix (10 mg IgG mL^−1^) was defined for the third milking. As the number of milkings increases, the IgG concentration in the colostrum decreases, such that the detection of the IgG concentration also becomes increasingly difficult. At low IgG levels, the lower detection limit is reached. When the IgG concentration is this low, colostrum should no longer be used for the first feeding of newborn calves. According to the calculated sensitivities (0.51) and the area under the curve (0.51), the authors consider refractometers to no longer be valid as of the fourth milking. In the fifth milking, an IgG concentration of only 10 mg mL^−1^ was found. Based on their results, the authors recommend using a digital refractometer for the first, second, and third milkings postpartum [53].

Refractometers are break resistant and only require a few drops of colostrum to perform measurements [10,50,51]. All in all, they are cheap (25.00–200.00 €, German trade prices) and a quick tool that can be used with little additional equipment or training [8,47]. Nevertheless, the refractive index of milk and colostrum depends on the concentration and composition of the total solids. More specifically, the volume and distribution of protein in the colostrum, as well as the fat content and casein micelles, affect the accuracy of measurements taken using refractometers [56]. As such, higher correlations cannot be achieved with respect to the gold standard (RID).

#### 2.2.2. Colostrometer

Another tool to estimate the Ig concentration in colostrum is a colostrometer (hydrometer). The colostrometer consists of a measuring cylinder, spindle, and a float, allowing conclusions about the specific gravity due to its displacement. The density correlates with the Ig concentration in the colostrum. Based on this correlation, the density measured with the colostrometer can conclude the Ig concentration. The float contains a scale of different colored areas indicating three different levels of Ig concentration in colostrum (green: >50 mg Ig mL^−1^, yellow: 20–50 mg Ig mL^−1^, red: <20 mg Ig mL^−1^) [57].

The colostrometer was first described by Fleenor and Stott (1980) [57], who showed the linear relationship between the total Ig concentration and the specific gravity of colostrum.

Bartens et al. (2016) [10] tested two hydrometers from different companies for accuracy and precision in measuring IgG in colostrum with regard to the optimum sample temperature (20 vs. 37 °C). The cut-off points specified by the manufacturers for “good” colostrum (>50 mg IgG mL^−1^ obtained with RID) were 1.047 and 1.045 for the two different hydrometers adapted to 20 and 37 °C, respectively. Furthermore, the optimal cut-off points were determined independently of the manufacturers’ specifications. An optimized cut-off point of 1.055 was evaluated using a receiver operating characteristic curve for the hydrometer used at 20 °C and 1.054 for the hydrometer used at 37 °C [10]. In another study, different cut-off points for the colostrometer were tested to detect colostrum containing 50 mg IgG mL^−1^. Specificity, accuracy, sensitivity, positive predictive value, and negative predictive value were compared. The highest combined sensitivity and specificity for detecting adequate colostrum defined using RID occurred at a cut-off point of 80 mg IgG mL^−1^. The sensitivity was 0.84 and the specificity was 0.77. At this cut-off point, the colostrometer had an accuracy of 0.80 [7]. In contrast to the previously described study, Chigerwe et al. (2008) [1] determined an optimal cut-off point for two different hydrometers. For the first colostrometer, cut-off points were investigated in ten steps from ≤10 to ≥140 mg mL^−1^. At the optimal cut-off point of 70 mg mL^−1^, the sensitivity and the specificity were 0.75 and 0.78, respectively. For the second colostrometer, they surveyed cut-off points in steps of 12.5 from ≤25 to ≥125 mg mL^−1^. An optimal cut-off point of 87.5 mg mL^−1^ was calculated, which was achieved at a sensitivity and specificity of 0.75 and 0.66 [1]. The authors stated that instrument-specific cut-off points should be defined within the scope of these variations, even with the same instruments. For the first colostrometer, they recommend a range of 60 to 90 mg mL^−1^ for possible cut-off points. The second colostrometer should have a range from 75 to 100 mg mL^−1^ [1]. Table 4 summarizes the different sensitivities, specificities, and correlations of the colostrometer studies.

In a study by Bartens et al. (2016) [10], the second utilized hydrometer had a similar sensitivity but a lower specificity than the first hydrometer. Based on these values, the accuracy for hydrometer one was higher than for hydrometer two. The authors stated that hydrometer one could be used directly after milking, whereas the colostrum for hydrometer two had to be cooled down first. They speculated that the results would have differed if both hydrometers had been used at the same temperature [10]. In 1991, Mechor and Gröhn [61] investigated the influence of temperature on the results of colostrometer readings. They collected 25 colostrum samples from Holstein-Friesian cows and measured the Ig concentration using a colostrometer. The colostrum temperature was increased in 5 °C steps from zero to 40 °C and the Ig concentration was measured at each step. They found a significant effect (*p* < 0.01) of temperature on the readings. The readings varied by 0.8 mg mL^−1^ between temperature levels. The regressions coefficients for colostrum and the sample temperature tended to rise with the increasing concentration category. The effect of the sample temperature on colostrometer results depends on the concentration [61].

The relationship between the IgG concentration in colostrum measured using a colostrometer and a refractometer has been assessed in some studies. Morrill et al. (2012) [44] confirmed the correlation determined, by Hassan et al. (2020) [60], for the IgG concentration measured using a refractometer and a colostrometer.

However, a colostrometer depends on the ambient temperature and the results are only comparable at a colostrum sample temperature of 20–21 °C [52]. Compared to the refractometer, the colostrometer is not break resistant and challenging to clean but cheaper (20.00–30.00 €, German trade prices) [10,62]. The results of the colostrometer also depend on the dry matter content, where a higher solid content or more fat in the colostrum leads to higher specific gravity [52].

#### 2.2.3. Split Trehalase IgG Quantification Assay and Zinc Sulfate Turbidity Test

Drikic et al. (2018) [27] tested a split trehalase IgG quantification assay (STIGA) for the measurement of IgG in colostrum. The results were compared with the gold standard RID and its potential as an on-farm tool was described. A STIGA is based on the enzyme trehalase (TreA), which converts trehalose into glucose. TreA splits into two non-functional fragments (TreA^N^ and TreA^C^). The fragments fuse with protein-G, which specifically binds to IgG and, thus, it acts as a sensor for IgG. If the fusion proteins are incubated with colostrum, binding with the IgG contained in the colostrum occurs. TreA is reactivated and glucose is formed from trehalose. The glucose formed can be detected with a colorimetric assay or a glucometer. Based on the glucose, the IgG concentration in the colostrum can then be indirectly inferred [27,63].

Dirkic et al. (2018) [27] performed a colorimetric assay (STIGA) and a glucometer test strip-based assay (STIGA^FIELD^). The authors found a correlation of IgG concentration for dairy breed colostrum measured using RID and a STIGA of r = 0.72. The correlation for beef colostrum was similarly high at r = 0.73. The highest sensitivity and specificity for dairy breeds were found at an optical density cut point of 0.9. For colostrum from beef breeds, the highest sensitivity (0.83) and specificity (0.90) were recorded at an optical density of 0.8. The STIGA identified 23% of the dairy colostrum samples as poor, whereas RID recognized 28.3%. Of the beef cow samples, 23.4% were defined as poor using a STIGA and 18.8% using RID. The correlation between the IgG concentration measured with RID and glucose concentration measured via glucometer (STIGA^FIELD^) is r = 0.7 for dairy colostrum and r = 0.94 for beef colostrum.

Compared to the indirect methods, a refractometer and a colostrometer, the STIGA shows a comparable sensitivity and improved specificity. The STIGA needs 90 min until a result is available. The authors also point out that fewer laboratory utensils are needed, and that the procedure can be automated [27]. In addition, a user-friendly method (STIGA^FIELD^) was tested, which, according to the authors, could also be used on farms. This variant does not require laboratory equipment or trained personnel.

Furthermore, strong correlations between the STIGA^FIELD^ and RID were determined. Therefore, the authors consider it a promising method to be tested under practical conditions [27]. A glucometer test strip-based assay was used to determine the concentration. This assay is commercially available for less than 25 € per test and represents a cost-effective variant.

In the zinc sulfate turbidimetry (ZST) test, salts are formed by chemical combinations of heavier globulins and trace metal ions. The salts precipitate and the interpretation can be made visually or with spectrophotometry. Visually, the test can be performed within 30 to 60 min. The concentration of IgG is proportional to turbidity. Even though a spectrophotometer is more precise, it takes longer to perform such a measurement. The measured optical density is compared with a standard curve [64].

Dunn et al. (2018) [35] tested ZST to approximate the IgG concentration in serum samples of ten Holstein-Friesian and ten Limousine × Holstein-Friesian cows. They found a significant (*p* < 0.001) positive correlation between the IgG concentration in serum samples measured using RID (R^2^ = 0.78) as well as the ELISA (R^2^ = 0.77) and IgG concentration measured using ZST. Pompermayer et al. (2019) [65] tested the ZST in practice and the laboratory to detect an FPT in foals. Although blood rather than colostrum was tested for the IgG concentration, conclusions about the practicality of ZST are possible. The ZST test was stored at the farm at room temperature, which varied considerably within the experiment (−1.2 °C to 32.3 °C). For comparison, a ZST test and a RID test were also performed in the laboratory. The number of false positives in the ZST on-farm tests was five times higher than in the laboratory samples.

The authors attribute this primarily to the difference in temperature, as ZST is temperature dependent. The study calculated a correlation of 0.92 (*p* < 0.0001) between the temperature and the turbidity of the zinc sulfate solution after reaction with serum. They suggest that the low temperature slows down the reaction [65]. The strong temperature dependence should be considered negative for practical use since the number of false-positive results should be kept as low as possible. The authors suggest warming the blood to 30–37 °C. However, these findings should be confirmed regarding the IgG concentration in the colostrum samples to obtain more precise data. The practical use, especially concerning temperature, should be further considered in future studies. In addition, the cost of a spectrometer is very high at up to 2000 €. Turbidity can also be assessed manually, but this assessment is inaccurate.

#### 2.2.4. External Characteristics

Colostrum comes in a variety of colors, ranging from dark brown to yellow to white. The color of the colostrum is often linked to its Ig concentration, with a lighter milk signifying a lower density [66]. The same applies to viscosity, the flow resistance of a liquid, which is often used as an indirect indicator of the Ig concentration. It has long been assumed that colostrum with a higher viscosity has a higher concentration of Ig. Due to that, viscosity has been measured visually for a long time as an indicator of the colostrum Ig concentration. The simplest method is the visual assessment of the flow properties, although this is the least accurate method. There are measuring instruments that can assess or directly measure the viscosity of the colostrum. However, within the scope of this review, only a few studies were found that examined the relationship between viscosity and Ig concentration in colostrum [47].

Different viscometers can determine viscosity; this includes, for example, an outlet funnel [5,47]. When using an outlet funnel, the time until a defined volume of colostrum runs out entirely is stopped. Based on the transit time, the viscosity of the colostrum can be inferred [5]. An outlet funnel costs around 15.00 €. Kritzinger (2017) [5] demonstrated, in his study with 124 Simmental cows, a positive correlation (r = 0.42) between funnel run time and IgG concentration in colostrum measured using RID. According to his results, colostrum (100 mL) with a transit time longer than 24 s should indicate an IgG concentration of >50 mg mL^−1^. The specificity and sensitivity of the method were 0.78 and 0.74, respectively.

Hassan et al. (2020) [60] found a significant correlation (r = 0.58; *p* < 0.05) between the viscosity measured using an electronic viscometer (dynamic viscosity) and the IgG concentration in colostrum determined using the colostrometer. A significant correlation of r = 0.74 (*p* < 0.05) was obtained compared with a digital refractometer. In addition to determining the viscosity using an electronic viscometer, the viscosity was also assessed visually, and the colostrum was divided into the following categories: watery, liquid, and thick. Significant correlations were found between the visual viscosity and the IgG concentration measured using the digital refractometer and the colostrometer. For the concentration in mg mL^−1^ estimated using the colostrometer and the visual viscosity, the correlation was r = 0.90 (*p* < 0.05). The correlation between the Brix value and dynamic viscosity is given as r = 0.84 (*p* < 0.05). A significant correlation (r = 0.63; *p* < 0.05) was also found between the visual viscosity and the dynamic viscosity [60]. Another study showed no correlation regarding the IgG concentration and the liquidness or thickness of the colostrum [58].

Chigerwe et al. (2008) [1] considered the colostrum yield to indicate a sufficient concentration of IgG. They used a digital scale to determine the amount of the first milking in 171 cows. The mean colostrum weight of the first milking was 7.4 ± 3.9 kg. The cut-off point calculated by sensitivity (0.42) and specificity (0.74) for the determination of colostrum with <50 mg mL^−1^ is given as 8.5 kg. With this cut-off point, 56 of the 171 colostrum samples were classified as adequate. Due to the low sensitivity, the authors do not recommend the weight of colostrum as a suitable indicator for colostrum with sufficient IgG [1].

**Figure 1:**
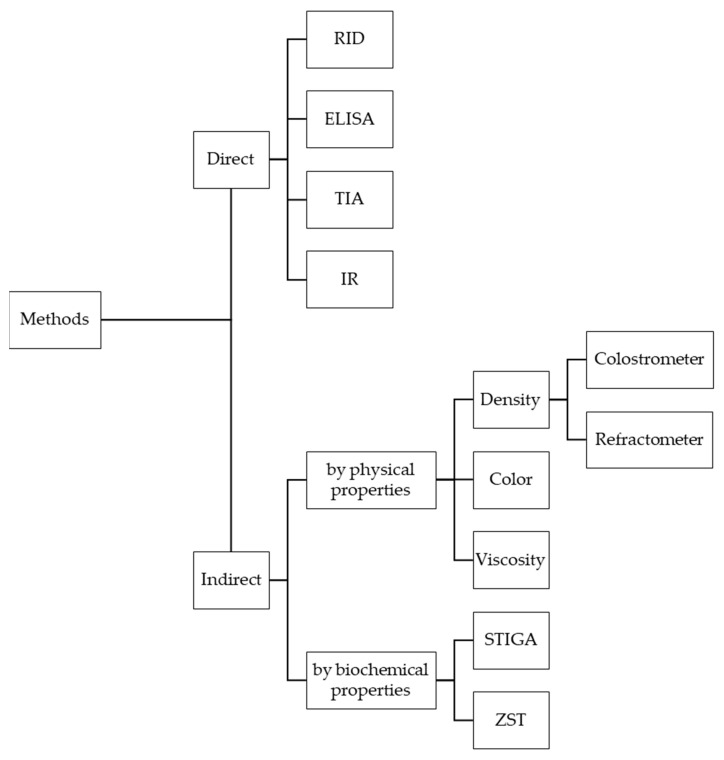
Overview chart of the direct and indirect methods for determining Ig concentration in bovine colostrum presented in Section 2.1 and Section 2.2 (RID—Radial immunodiffusion; ELISA—Enzyme-linked immunosorbent assay; TIA—Turbidimetric immunoassay; IR—Infrared; STIGA—Split trehalase IgG quantification assay; ZST—Zinc sulfate turbidity test).

A study by Gross et al. (2014) [66] demonstrated the relationship between colostral IgG concentration and color measurement for 117 colostrum samples from Holstein-Friesian cows. No significant correlation (r = −0.08; *p* = 0.40) could be found between the color measurement and the IgG concentration. The lactation did not influence the relationship between the two parameters. To classify colostrum into high- and low-quality, the following three threshold values were set: 50, 75, and 100 mg IgG mL^−1^. The highest sensitivity (0.50), specificity (0.50), and negative predicted value (0.88) were calculated at the threshold of 50 mg IgG mL^−1^. According to the authors, color measurement is a method to conclude the IgG concentration. However, the inference of IgG concentration with the visually perceived colorfulness (chroma value G) is insufficient and does not improve over other instruments, such as the refractometer [66]. The color of the colostrum is a very subjective factor for concluding the IgG concentration of the colostrum [58]. If the assessment is performed visually without technical support, the result depends heavily on the performing person and their experience. Therefore, farmers should not rely solely on this assessment when providing calves with sufficient colostrum. By using measuring devices such as a spectrophotometer, the color measurement could provide an additional way of determining the Ig concentration in the future, next to the colostrometer and refractometer [66]. Figure 1 gives a final overview of the measurement methods and tools described in this review.

### 2.3. Dissemination of the Methods

The control of colostrum quality, i.e., the determination of Ig concentration, does not seem to be widespread in dairy farming. In a survey conducted by Klein-Jöbstl et al. in 2015 [26], 1287 Austrian farmers participated and 78.7% stated that they do not verify the Ig concentration in colostrum before feeding. Only 20.8% of the respondents tested the Ig concentration in the colostrum on their farms, and 0.5% did not answer the question. The test is mainly performed by visual observations (86.1%) [26]. In a survey from Germany, 92.9% of the respondents (*n* = 42) reported controlling colostrum intake; however, only 23.8% noted the Ig concentration of the colostrum [67]. The result is similar to the Austrian study.

## 3. Factors Associated with Ig Concentration in Colostrum

The variation of Ig concentration in colostrum is high and influenced by several factors. For optimal colostrum management, it is helpful to know which factors influence the Ig concentration and to what extent. It is also important to understand how these parameters can be employed to improve the Ig concentration in colostrum. To feed calves colostrum with a high Ig concentration, the reducing factors that lower the Ig concentration should be avoided as far as possible. A distinction can be made between animal-related and environmental-related factors. In the following, the factors and their influence will be examined in more detail.

### 3.1. Animal-Related Factors

#### 3.1.1. Colostrum Yield

In their studies, Silva-Del-Rio et al. (2017) [68] and Cabral et al. (2016) [69] demonstrated a negative correlation between colostrum yield and IgG concentration. The negative correlation of r = −0.42 in Cabral et al. (2016) [69] is slightly higher than in Silva-Del-Rio et al. (2017) [68], who calculated a negative correlation of r = −0.37. The IgG concentration in the colostrum decreased with an increasing colostrum yield [68]. Kehoe et al. (2011) [59] also determined a negative but weak correlation of r = −0.16. Furthermore, the regression analysis showed a relationship between the colostrum yield and the IgG concentration (R^2^ = 0.03; *p* < 0.01) [59].

Scholz et al. (2011) [70] concluded that a first milking quantity of more than 7.2 L negatively affects the Ig concentration. Cows with more than 7.2 L of colostrum at first milking had both the lowest total protein content (35–205 mg mL^−1^) and the lowest Ig concentration (14–179 mg mL^−1^). Cows with less than 4.5 L of first milk had a total protein content of 38–245 mg mL^−1^ and an Ig concentration of 20–203 mg mL^−1^ [70]. Løkke et al. (2016) [62], determined a correlation of 0.70 between the total protein content and the IgG concentration. In a study with Holstein-Friesian cows, an increase in the colostrum volume by 1 L showed a 1.4 mg mL^−1^ lower Ig concentration [71]. Another study found no influence of the milked colostrum quantities on the IgG concentration in the colostrum [5].

The decreasing colostrum Ig concentration depends on water diffusion. When lactation starts, the secretion of lactose into the udder increases, whereas the absolute amount of IgG remains the same. Due to the higher volume, there is more dilution [69,72].

#### 3.1.2. Parity

According to Ganz et al. (2018) [73], the Ig concentration in colostrum is correlated with the number of lactations. Older cows produce colostrum with higher Ig levels; this may be because older cows have been exposed to antigens for a longer time than younger cows. Antibodies transfer from the mother cow’s serum to the colostrum. As a result, parity can positively influence the Ig concentration in colostrum [74].

Kehoe et al. (2011) [59] determined a notably increased IgG concentration in the colostrum from cows in lactations one to four. Furthermore, cows in the second lactation produced colostrum with the lowest IgG concentration compared to cows in all other lactations; however, there was no statistically significant difference between the first and second lactation cows [59]. This result can also be attributed to a dilution effect. Older cows have been exposed to various antigens over a longer period [74]. On the one hand, cows in the second lactation have not been exposed to the environment for a substantial period, but, on the other hand, they do produce significantly more milk than those in the first lactation [75]. As such, the lower concentration of Ig is more diluted in colostrum from the second lactation, compared to that from the first lactation, which may explain the lowest IgG concentration in colostrum from second lactation cows.

**Figure 2:**
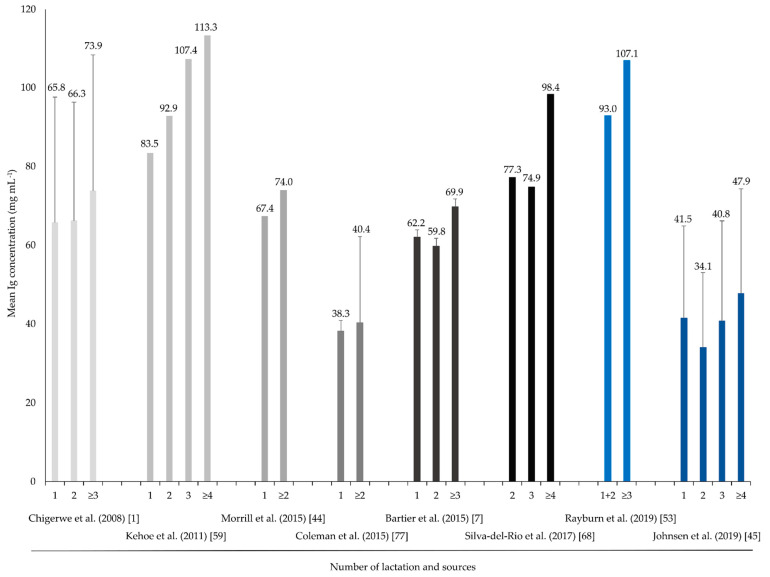
Mean Ig concentration (mg mL^−1^) of cows in different lactations (L = Lactation) [1,7,44,45,53,59,68,77].

In a study of Norwegian dairy cows, Gulliksen et al. (2008) [76] noted an increase in the IgG concentration as the lactation number increased; this increase was particularly evident between cows in their first or second lactation and cows in their fourth or greater lactation. Figure 2 shows the Ig concentrations collected across different studies with respect to the lactation number.

Muller and Ellinger (1981) [78] noted a lower IgA concentration (*p* < 0.05) in colostrum from cows in the first lactation compared to those in the third or fourth lactation. When the total Ig concentrations were compared across lactations, cows in the third and fourth lactations had higher levels than cows in the first lactation [78].

Additionally, the mean IgG concentration is often considerably higher during the third lactation than in the first or second lactation [1,30,53,59,68]. This finding was confirmed by Scholz et al. (2011) [70], who noted that the total protein content of young and two-calf cows was significantly (*p* ≤ 0.05) lower than that of higher parity cows. The Ig concentration of young and second calf cows was also lower (*p* ≤ 0.05) than that of higher parity cows [70]. Similarly, Phipps et al. (2017) [79] found that cows in the fourth or higher lactation had the highest mean Ig concentration in their colostrum in contrast to lower parity cows. More specifically, 49.3% of cows in the fourth lactation had an IgG concentration greater than 50 mg mL^−1^, whereas only 27.9% of cows in the second lactation reached this level. The authors attributed this result to an increased colostrum volume, compared to other lactations, and a stronger dilution effect. Additionally, Silva-Del-Rio et al. (2017) [68] tested the IgG concentration in the second milking of cows. As previously described, the IgG concentration in the second milking is higher in cows in the fourth or greater lactation, compared to those in the second or third lactation [68]. Similar results were described in a study by Johnsen et al. (2016) [45].

Other studies show a weak or no correlation between the lactation number and the colostrum Ig concentration [77,80,81]. Cabral et al. (2016) [69] determined a weak correlation (r = 0.22) between the number of lactations and the IgG concentration in the colostrum of Holstein-Friesian cows [69]. Morrill et al. (2015) [44] and Coleman et al. (2015) [77] observed no differences in the colostrum IgG concentration between primiparous and multiparous cows, based on standard methods (RID and TIA). Conversely, Morrill et al. (2015) [44] noted that multiparous cows had a higher IgG concentration than primiparous cows, according to measurements taken with a refractometer and a colostrometer.

In most studies, increases in Ig concentrations are dependent on the number of lactations and do not start until the third lactation. Therefore, it is difficult to make conclusions about animals in the second lactation since they are integrated into the multiparous group. Secondly, biased by milk yield, the effect of lactation alone cannot be evaluated, and colostrum can be classified as good or poor quality by the lactation number alone. Due to the differences between cows in different lactations, colostrum from primiparous cows should only be fed to calves after determining its IgG concentration; it should be replaced by colostrum with a higher Ig concentration if necessary [59,70]. Primiparous cows usually have a low IgG concentration, which can lead to an FPT. Colostrum with low IgG concentrations can be flagged via measurements, such that colostrum with a higher IgG concentration can be used instead; this has a positive effect on the immune status and, thus, the development of the calf. In this context, different measurement cut-off points should be defined in relation to the number of lactations. According to Bielmann et al. (2010) [20], it is unnecessary to define different cut-off points according to the lactation number when making measurements with a refractometer. A cut-off point of 22% Brix can be used for colostrum from first-calf heifers and cows in higher lactations.

Studies have shown that the leakage of colostrum from the udder influences its Ig concentration [82]. In a study by Reschke et al. (2017) [82], colostrum leakage was the most significant (*p* < 0.001) risk factor for the production of colostrum with an insufficient Ig concentration. Regardless of whether the cow loses colostrum prior to or during birth, this loss has a negative effect on its Ig concentration. The IgG-rich colostrum in the udder at the end of the dry period is thus lost [4]. In the case of colostrum being lost through leakage, Ig concentrations shift earlier to transition milk; however, this milk is excreted at the time of the first colostrum. In practice, farmers observed that colostrum leakage appears more frequently in cows with higher lactation numbers, but data on leakage at early lactation are rare. Leakage appears to occur in comparable proportions in multiparous and primiparous cows starting from day nine [83].

#### 3.1.3. Breed and Genetic

Comparative studies suggest that there are differences in the Ig concentration in colostrum between different breeds. In 1981, Muller and Ellinger [78] already investigated the Ig concentration in the colostrum of five different cattle breeds. They analyzed colostrum samples from Ayrshire, Brown Swiss, Guernsey, Holstein-Friesian, and Jersey cows using RID. No significant differences could be found about the individual Ig concentration. However, a trend became apparent in that Jersey cows consistently had the highest IgG, IgA, and IgM concentration in the colostrum. In terms of the total Ig concentration, Jersey and Ayshire cows had higher values than Holstein-Friesian, Guernsey, or Brown Swiss cows [78]. The mean IgG concentration for Jersey cows in Morrill et al. (2015) [44] was 72.81 mg mL^−1^ and had a range of 12.82 to 154.26 mg mL^−1^, whereas the values in Silve-Del-Rio et al. (2017) [68] were slightly higher (83.5 mg mL^−1^, 23.7–172.9 mg mL^−1^). The mean IgG concentration for Holstein-Friesian cows is similar but mostly lower compared to Jersey cows, with 68.5 [1], 64.7 [11], 65.1 [42], 57.65 [60], and 73.4 mg mL^−1^ [8]. In another study, the IgG concentration in the colostrum of Holstein-Friesian cows, Jersey cows, and a Holstein-Friesian-Jersey cross was determined using a Brix refractometer. For the Holstein-Friesian cows, the %Brix value was 18.9%, for Jersey cows it was 21.3%, and it was 20.1% for the crossbreds

Consequently, in this study, Jersey cows showed a higher IgG concentration than Holstein-Friesian cows. However, the factor breed failed to be significant (*p* < 0.05) [79]. A study with 2500 lactating Jersey cows recorded a mean Brix value of 26.6% [84]. These results are not congruent with the outcomes of Coleman et al. (2015) [77], who did not find differences between the concentration of IgG in colostrum from Holstein-Friesian and Jersey cows.

In general, beef cows should have a higher Ig concentration in the colostrum than dairy cows. This opinion is concordant with the studies of Gamsjäger et al. (2020) [54], in which the cut-off points for low-IgG colostrum and high IgG colostrum for 416 colostrum samples from one beef breed deviate strongly from the normally used cut-off point of 50 mg IgG mL^−1^. The cut-off point for low-IgG colostrum was at <100 mg mL^−1,^ and the cut-off point for high-IgG colostrum was at ≥150 mg mL^−1^. Although these values are much higher, 49.8% of the samples contained IgG ≥150 mg mL^−1,^ and only 9.1% were below the cut-off point of 100 mg mL^−1^. However, even in this study, the IgG concentration in the colostrum varied greatly (19.2–264.7 mg mL^−1^). These variations are also found in studies with dairy breeds [54]. The average IgG concentration measured using RID in a study by Elsohaby et al. (2018) [43], including beef cows, was 143.2 mg mL^−1^, just below the previously indicated cut-off point of 150 mg mL^−1^. This cut-off point was even exceeded for colostrum samples from Charolais in Martin et al. (2021) [6] (158.44 mg mL^−1^). In contrast to the IgG concentration in the colostrum of beef cows, an average IgG concentration of 65.5 mg mL^−1^ was found in a study by Elsohaby et al. (2018) [43] for dairy cows. Since it seems that beef breeds have higher Ig concentrations in their colostrum, the cut-off point for these breeds could be set directly higher than for dairy breeds. Considering the calf’s intake capacity and need, calves with a low intake should be fed with colostrum containing a high concentration of Ig. In this way, the calf is able to absorb a sufficient amount of Ig despite the low quantity of colostrum provided. Accordingly, the colostrum that exceeded a higher cut-off point could be used at this point.

In their study, Vandeputte et al. (2014) [81] measured the IgG_1_ concentration in the colostrum of four beef breeds (Charolais, Belgian Blue, Blonde d’Aquitaine, and Limousine). However, the average IgG_1_ concentration did not differ between the four breeds. The mean IgG concentration across all the breeds was 95.9 mg mL^−1^, which is higher than figures recorded in studies with dairy breeds [81].

Dunn et al. (2018) [35] did not detect any differences between the IgG concentration in the colostrum of ten Holstein-Friesian and ten crossbred animals (Limousine × Holstein-Friesian). In addition, the factor breed did not affect the IgG concentration, neither related to the first nor the fifth milking after birth [35]. However, the sample size is minimal, and the results should be evaluated accordingly.

**Figure 3:**
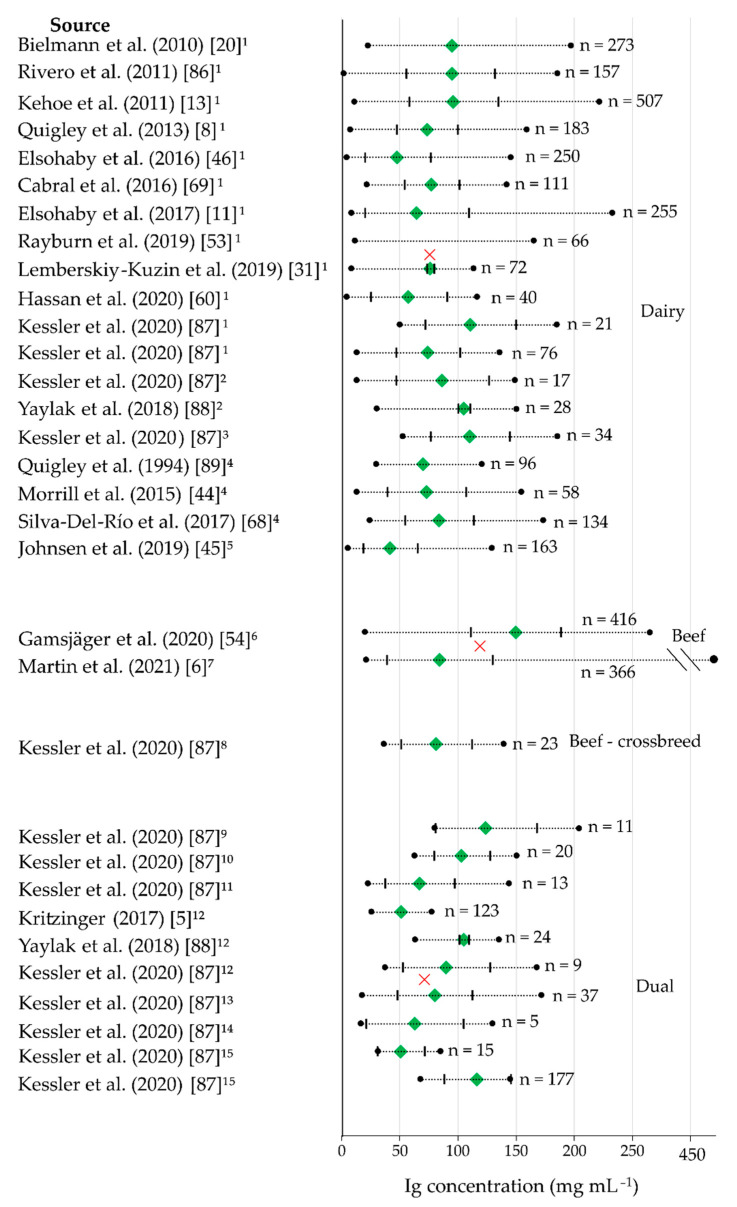
Ig concentration (mg mL^−1^) in colostrum of different breeds sorted by dairy, beef, meat crossbreeds, and dual breeds ● Minimum and Maximum Ig concentration, 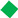 Mean Ig concentration, ❌ Minimum and Maximum SD,▐ Weighted average of the group. ¹ Holstein-Friesian; ² New Zealand Holstein-Friesian; ³ Brown Swiss; ⁴ Jersey; ⁵ Norwegian Red; ⁶ mixed beef breeds; ⁷ Charolais; ⁸ Holstein-Friesian × Charolais; ⁹ Montbéliarde; ¹⁰ Holstein-Friesian × Montbéliarde; ¹¹ Pinzgauer; ¹² Simmental; ¹³ Rhetic Gray; ¹⁴ Murnau-Werdenfelds; ¹⁵ Original Braunvieh. [5,6,8,11,13,20,31,44,45,46,53,54,60,68,69,86,87,88,89].

Figure 3 shows varying Ig concentrations of different studies, subdivided by the breeding goal of the used cows. The minimum and maximum Ig concentration, mean Ig concentration, minimum and maximum standard deviation (SD), and the weighted mean of the groups are shown. Specific breeds were ranked within the breed groups by the milk yield (high to low) they produced. It is suspected that the breed-specific differences are due to genetic parameters and dilution effects [15]. In a comparison between Holstein Friesian and Charolais, the concentration of IgG_1_ (*p* = 0.06), IgG_2_ (*p* < 0.01), IgM (*p* < 0.01), IgA (*p* = 0.08), and total Ig (*p* < 0.05) in colostrum were found to be higher in Charolais. In Holstein-Friesian cows, the total mass (concentration × yield) of IgG_1_, IgG_2_, IgM, IgA, and total Ig was significantly higher [85]. However, the colostrum yield produced by Holstein-Friesian cows is higher than that produced by Charolais [85]. It can be concluded that there is a greater dilution of Ig and, as a result, a lower concentration of Ig.

Some studies have explored the relationship between genetic aspects and the Ig concentration in colostrum. Karl and Staufenbiel (2017) [71] identified cow sires as an important antepartum influencing factor on the Ig concentration. For the authors, genetics, i.e., the cow sire, even represents the most important influencing factor on Ig concentration. In their opinion it is, therefore, possible to influence the Ig concentration in the colostrum of the daughters through targeted selection. However, they also pointed out that the bull’s daughters who inherited the highest Ig concentration also had the lowest colostrum yield at the first milking. This outcome indicates the dilution effect already mentioned. In addition, the individual range of the animals must still be considered. Nevertheless, the authors see genetics as a starting point for influencing the colostrum Ig concentration [71].

Conneely et al. (2013) [74] calculated a low heritability of 0.10 for the IgG concentration. The genetic standard deviation for IgG concentration and genetic variation coefficient were 16.0 mg mL^−^^1^ and 14.3%, respectively [74]. A study by Soufleri et al. (2019) [90] focused on the genetic background of the Ig concentration in colostrum and calculated the heritability for the total protein content and the colostrum total solids. Total solids in colostrum can be calculated indirectly using a refractometer and can be used to estimate the Ig concentration in colostrum. The total protein content had a heritability of 0.19 and total solids, a heritability of 0.27 (*p* < 0.05).

#### 3.1.4. Dry Period Length

Scholz et al. (2015) [70] found that the duration of the dry period has an influence on the Ig concentration in the colostrum. In their study, cows with a dry period longer than 62 d had 21 mg mL^−1^ more total protein content in their colostrum, compared to cows with a dry period of 46 d. In addition, a longer dry period (46 d) resulted in a 17 mg mL^−1^ higher Ig concentration in the colostrum. Furthermore, the total protein content in the colostrum varied considerably with the dry period length and number of lactations. The authors observed an increase in the total protein content of colostrum from second calving cows (from 61 to 93 mg mL^−1^) when the dry period increased from 46 to 62 days. However, the influence of the dry period length decreased from the fourth lactation onwards (*n* = 238) [70]. According to Cabral et al. (2016) [69], the IgG concentration is weakly, but positively, correlated with the dry period length (r = 0.17) and colostrum yield (r = 0.09).

These results were confirmed by Karl and Staufenbiel (2017) [71]. They found a significant correlation (r = 0.14, *p* < 0.05) between the Ig concentration and the dry period length. Extending the dry period by one day led to a 0.05 mg mL^−1^ increase in the Ig concentration, whereas a 10-day extension increased the Ig concentration by 2.2 mg mL^−1^. The authors stated that the regeneration of the udder during the dry period influences the Ig concentration in the colostrum. However, the authors also consider this influence to be too small in practice, as the dry period’s length is not only determined by the expected colostrum Ig concentration. Instead, the length depends on management factors and is a complex procedure [71,91]. Rastani et al. (2005) [92] noted a lower IgG concentration in cows without a planned dry period, compared to cows with a dry period of 28 d (49.8 vs. 77.9 mg mL^−1^). According to Watters et al. (2007) [93] and Gulay et al. (2005) [94], the IgG transfer into colostrum is not affected by a reduction in the dry period length. In colostrum samples from 781 Holstein-Friesian cows, there was a slight difference in the IgG concentration depending on the dry period length. One group of cows was dry stalled for 55 days, whereas the other group was dry stalled for 34 d; the former group had an IgG concentration of 5849 mg dL^−1^, and the latter group had a similar IgG concentration of 5616 mg dL^−1^ (*p* = 0.31) [93]. Mansfeld et al. (2012) [91] hypothesized that the decline in Ig concentration is due to the dilution effect that occurs if there is no dry period. The dilution effect leads to low IgG levels in the colostrum [91]. Colostrum is formed during the last weeks of gestation, and changes in oestrogen and progesterone concentrations have a decisive influence on the transportation of Ig into the milk. At the beginning of calving, transportation decreases due to rising prolactin levels; IgG transport is eventually terminated [5,95].

A longer dry period also occurs when the pregnancy lasts longer. With a longer gestation, the dry period is also longer; thus, IgG transport into the udder is possible over a longer period, leading to a higher concentration in the colostrum.

### 3.2. Environmental Factors

#### 3.2.1. Time from Calving to Milking and First Feeding Postpartum

Studies indicate that managing the interval from birth to first milking should be considered in terms of securing an adequate colostrum supply, especially on farms where the IgG concentration in colostrum is generally very low [68]. A study of 56 Holstein-Friesian cows assessed calving-to-first milking intervals of 0.3 to 23.8 h, noting a significant negative (R^2^ = 0.18; *p* = 0.001) relationship between the IgG concentration in colostrum and the interval between calving and first milking. The longer the time interval to the first milking is, the lower the IgG concentration is. In fact, the IgG concentration decreased by 3.7% with every increasing hour [14]. In a study by Kritzinger (2017) [5], the IgG concentration of the colostrum decreased by a factor of 1.7 every hour. The influence of the interval between calving and milking was described as statistically significant (*p* = 0.013) and the calculated correlation coefficient was −0.22 [5]. In agreement with the results of Sutter et al. (2019) [30], both studies described a negative correlation between the interval from calving to colostrum collection. Within a study by Scholz et al. (2011) [70], there was a 41% decrease in the Ig concentration in the first 9 h after birth. An additional study showed that the IgG concentration in the colostrum collected 6 h after birth was already lower (*p* < 0.05) than the concentration of the colostrum collected 2 h after birth [96].

A similar trend (i.e., negative correlation between the time to first milking and the IgG concentration) was also found when using a refractometer. When the first milking took place in the first 12 h postpartum, the average %Brix value was higher (24.4% Brix) than when the milking took place after more than 12 h (17.5% Brix; *p* < 0.05). More specifically, 68.6% of the samples obtained in the first 12 h were above or equal to 22% Brix (≥50 mg IgG mL^−1^); of the samples taken after the first 12 h, 16.3% met or exceeded this threshold. Overall, the %Brix value decreased by 25% per hour postpartum [79].

Elfstrand et al. (2002) [97] investigated the concentration of different Ig (IgG_1_, IgG_2_, IgA, and IgM) in colostrum collected in the first three to four milkings (from 0 to 80 h after birth) using RID. The concentration of all four Ig subtypes decreased as the number of milkings increased. IgA had the highest concentration (1.6 mg mL^−1^) in the first 10 h postpartum, which decreased by 50% in the next 10 h. IgG_2_ decreased by 30% in the first 10 h postpartum, but then remained unchanged in the following 10 h. The concentration of IgM decreased by half in the first 11 to 20 h postpartum; in the next 10 h, the concentration reduced by an additional 10%. The authors concluded that the concentration of individual Ig decreases with each milking; however, this occurs at different rates over the entire period, depending on the Ig subtype [97].

Table 5 shows the different concentrations at time points <6, 6–11, and >11 h postpartum, in comparison to the concentrations of IgG in the studies by Silva-Del-Rio et al. (2017) [68] and Moore et al. (2005) [96].

In a study by Silva-Del-Rio et al. (2017) [68], the average IgG concentration in the first milking (9 h 25 min, SD = 3 h 50 min) was 83.8 mg mL^−1^, whereas the average in the second milking (21 h, SD = 3 h 40 min) was 46.9 mg IgG mL^−1^ [69]. Rayburn et al. (2019) [53] also measured the IgG concentration in colostrum (first milking after birth) as well as in the second to sixth milking after birth using a cut-off point of >50 mg IgG mL^−1^ as indicative of a sufficient Ig concentration; 95.5% of colostrum samples exceeded the cut-off, and 36.5% of second milking samples were above the cut-off point. For the third milking after birth, the cut-off point was decreased to >25 mg IgG mL^−1^, and 13.1% of the samples exceeded this value. The cut-off point for the fourth and fifth milking after birth was >10 mg IgG mL^−1^ and 23.7 and 3.8% of the samples, respectively, reached the cut-off point. From the sixth milking onward, all the samples had an IgG concentration of less than 10 mg IgG mL^−1^. The samples from the third milking onward had lower IgG concentrations than those in the colostrum and second milk samples. As such, feeding a calf milk from the third milking after birth and onward would lead to a lower IgG intake and an FPT may occur [53]. However, the third and fourth milkings should still be included in the calf’s diet because the intake of IgG over a longer period after birth reduces the incidence of diarrheal diseases [98]. Colostral Ig acts in the blood serum but can still have a local protective function in the digestive tract after intestine closure [99]. As such, the prolonged feeding of colostrum can lead to reduced morbidity in newborn calves and reduced use of antimicrobials on farms [98].

Based on the results of these studies, the early milking of cows after birth is clearly necessary to obtain colostrum with high Ig levels. All the studies show a clear decreasing trend for Ig concentration with increasing distance to calving. Implementing this management practice is the only way to ensure an adequate supply of Ig for the calf [14,70].

The importance of the adequate and timely supply of colostrum is well understood. In a survey of 92 participants in Germany, 95.1% of respondents stated that the fastest possible supply of colostrum is the most important aspect of colostrum management [100]. Additionally, 83.7% of the respondents of an Austrian survey feed the first colostrum to the calf within the first 4 h of life, with 13.5% providing it within 4 to 6 h after birth; only 1.1% feed the first colostrum later than 6 h after birth. Most respondents feed around 2 to 4 L of colostrum in the first 6 h of life (71.9%); however, 13.3% of respondents feed less than 2 L. On the other hand, 12.7% of respondents feed more than 4 L to their calves in the first 6 h of life [26]. In another study, 72.5% of respondents (*n* = 40) reported that the first feeding of colostrum occurs within the first 6 h of life; however, 72.5% feed restrictively, 27.5% feed ad libitum, and 35.0% feed a minimum of 3.0 L [67]. These studies illustrate that calves should be fed colostrum as soon as possible after birth. The first feeding should take place within the first 2 h after birth [15]. Accordingly, the first milking should also occur within this period, although Godden et al. (2008) [4] noted that a delay of up to 6 h was acceptable.

If the colostrum contains a high concentration of Ig, the volume of colostrum that needs to be fed may be lower than if colostrum has a lower Ig concentration. In the latter case, the calf has to take in more colostrum to absorb the same amount of Ig [52]. In this respect, the maximum voluntary intake of each calf should also be considered; not all calves have the same intake and forced feeding (e.g., via a tube) can have negative consequences for the calf, such as gassing of the rumen. Overfeeding must, therefore, be avoided [101].

#### 3.2.2. Treatment Procedures

To ensure a timely supply of calves with colostrum that has a sufficient Ig concentration, it is recommended that frozen colostrum reserves are kept. These reserves can be used if fresh colostrum is unavailable in time or if the dam’s colostrum does not contain enough Ig and an FPT could occur. Before feeding, the frozen colostrum must be thawed and warmed up gently but also quickly. To feed adequate colostrum, freezing, thawing, and heating processes must be known to influence the Ig concentration.

Morrill et al. (2015) [44] investigated the influence of freezing on IgG concentration in colostrum. The IgG concentration in the colostrum was measured using RID, a refractometer, and a colostrometer no later than 2 h hours after milking, and the colostrum was then frozen at −20 °C. After seven days, the colostrum was thawed for the first time and warmed to room temperature. Two further cycles followed. The samples were thawed and warmed again after 14 days and one year and the IgG concentration was measured at the respective time points. If the colostrum was frozen only once, no influence on the concentration was found compared to the measurement 2 h after milking using a refractometer and a colostrometer. After two freezing cycles, a lower IgG concentration was measured using RID in the colostrum. No difference was observed if the cow was primiparous or multiparous. An influence on the results of the refractometer and colostrometer measurements was also excluded. The authors suggested that the multiple freezing cycles have a negative impact on the accuracy of the RID [44]. In a study by Bielmann et al. (2010) [20], high correlations were found between the Brix values measured using optical and digital refractometers for fresh and frozen colostrum samples (r = 0.98 and r = 0.97; *p* < 0.001). According to the authors, these results showed that the freezing and steeping of colostrum do not influence the results of the two measuring devices [20]. Furthermore, heating colostrum does not seem to affect the Ig concentration of the results from optical and digital refractometers, regardless of the heating period or temperature [42].

Pfeiffer et al. (2010) [102] tested two different methods for thawing colostrum samples—water bath and microwave. In the water bath, the samples were thawed at 46 °C within 60 min and then heated. The microwave thawed the samples at 250 watts for 15 min under temperature control. The IgG concentration was determined for the fresh samples and the warmed samples using RID. Before heating, the mean IgG concentration of the samples was 138 mg mL^−1^. After heating, the mean IgG concentration was 79 mg mL^−1^ for the water bath samples and 76 mg mL^−1^ for the microwave samples. A loss of 44% was observed for both methods. The IgG concentration was, therefore, still above the limit of 50 mg IgG mL^−1^. No significant differences in the IgG concentration could be found between the two methods after thawing, although macroscopically visible coagulation was observed in the heated microwave colostrum. The authors concluded that thawing by microwave at 250 watts for 15 min has no negative effect on the IgG concentration of the thawed colostrum. Thus, this method approves to be a faster way of thawing, as the effort is reduced to 45 min compared to the water basin [102]. However, the authors’ conclusion should be considered critical because even when the Ig concentration is above 50 mg mL^−1^, there was a 44% loss. Since the Ig are a very valuable component of the colostrum, the loss should be kept as low as possible. The Ig concentration of the colostrum should be as high as possible, and losses should preferably not occur at all or only to a minimal extent. In this context, a reduction of 44% is certainly to be considered critical, even if the cut-off point has not yet been undershot. Larger surfaces can also be defrosted more quickly. This aspect could be considered in the freezing process. Furthermore, the vessel in which the colostrum is frozen could influence the thawing process. However, up until this date, studies on this are not available.

Elizondo-Salazar et al. (2010) [103] studied the identification of the ideal time and temperature range for heat treatments of colostrum with the least possible effect on the IgG concentration measured using RID. They found that the total IgG concentration decreases with increased temperature and over the time during which the colostrum was heated. A reduction was observed when the colostrum was heated to 60 °C, even if it was only heated for 30 min. The most significant decrease in the IgG concentration occurred at a temperature of 63 °C [103]. Elsohaby et al. (2018) [42] came to similar conclusions, where the average IgG concentration measured using RID was 45.6 mg mL^−1^. When the colostrum was heated at 63 °C for 30 min or 63 °C for 60 min, the average IgG concentration measured using RID decreased to 31.1 and 30 mg mL^−1^, respectively. The IgG concentration decreased by 27 and 29% [42]. Hassan et al. (2020) [60] treated colostrum at 60 °C for 60 min, at 63.5 °C for 30 min, and at 72.0 °C for 15 s in a water bath to find out which temperature cut-off point has an influence on viscosity in relation to IgG concentration. For all three temperature–time combinations, they found a change in the viscosity of the samples measured visually and using a viscometer. The authors conclude that heating colostrum (containing an IgG concentration lower than 80 and 68 mg mL^−1^) at 60 °C for 60 min and at 63.5 °C for 30 min has no significant effect on the viscosity or the IgG concentration independent of the measurement method [60]. An older study investigated a gentler heating process in which the colostrum was first heated to 60 °C for 30 min and held at this temperature for a further 120 min. After that, the colostrum was cooled down to 38 °C within 15 min. No difference in the IgG concentrations measured using a TIA was found between the fresh colostrum and the colostrum heated at 60 °C for 120 min. The reduction in IgG concentration was 2.2% [104].

Heating causes denaturation of the proteins, which results in their loss of regular activity [105,106]. The measuring methods that provide information on the Ig concentration via density (e.g., colostrometer and refractometer) cannot distinguish between intact and denatured Ig. Only specific methods (e.g., ELISA and RID) can do this. Therefore, if the effects of heating and freezing on the Ig concentration in colostrum are to be investigated, specific methods should be used for determination.

### 3.3. Other Possible Influencial Factors

Gross et al. (2017) [32] investigated the Ig concentration in colostrum at quarter-milking levels, in comparison with the Ig concentration of composite colostrum. There was no association between the colostrum quantity and the IgG concentration, whether at the quarter-milk level or within the composite colostrum. In their study, the concentration and total IgG mass in composite colostrum were higher in multiparous than in primiparous cows (*p* < 0.05), but there were no differences between primiparous and multiparous cows at the quarter-milking level. The range in values for IgG concentrations at the quarter-milking level was similar for primiparous and multiparous cows. In contrast, the IgG mass at the quarter-milk level was lower for primiparous cows than for multiparous cows [32].

In terms of somatic cell counts (SCC), cows with an SCC >50,000 cells mL^−1^ have lower IgG concentrations (<30 mg mL^−1^) after calving than cows with a lower SCC. There is no correlation between the SCC of the previous lactation and the IgG concentration [76]. Kehoe et al. (2007) [13] recorded higher IgG_2_ concentrations for cows on farms with a herd average SCC <200,000 cells mL^−1^ in the month prior to sample collection. Overall, the colostrum had a qualitatively higher nutrient composition at lower SCCs. These results contradict those of Cabral et al. (2016) [69], who did not detect any influence of SCC on the IgG concentration in colostrum. The SCC of the previous lactation has no effect on the IgG concentration; however, mastitis during the dry period can affect the IgG concentration in colostrum [69]. Furthermore, there was no correlation between common diseases (e.g., milk fever, prolonged pregnancy, retained placenta, dystocia, and mastitis) and the IgG concentration in colostrum [76].

Calving season may also have an influence on Ig concentration. Gulliksen et al. (2008) [76] noted a significantly (CI: 95%) lower IgG concentration in the colostrum from cows that calved in the winter, compared to other seasons. More specifically, cows calving in August, September, or October produced colostrum with higher IgG concentrations than cows calving in the other months. The authors assumed this was due to the advantage of the pasture, which is legally prescribed in Norway [76]; however, in a study by Pritchett et al. (1991) [107], there was no significant effect of season on the IgG_1_ concentration in colostrum. Farmers also suggest changing the stable environment to enhance immune responses and possibly the active Ig content, but there is no scientific evidence to support this strategy. In different seasons, there can be strong temperature fluctuations. According to Cabral et al. (2016) [69], heat stress has a negative effect on IgG concentration; they found a negative correlation between the number of days above 23 °C during the last 21 days before birth and the IgG concentration in the colostrum. An Italian study confirmed this negative correlation, wherein the concentration of IgG and IgA decreased under heat stress [108]. Conneely et al. (2013) [74] also found that cows calving in April produced colostrum with a lower IgG concentration than cows that calved in the early spring or fall. These studies illustrate the potential influence of environmental temperature on the IgG concentration in colostrum. This factor should be taken into account in future studies.

Blecha et al. (1981) [109] studied the effect of dietary protein restriction during the 100 days before birth on the Ig concentration in colostrum; they found no significant correlation between the concentrations of IgM, IgG_1_, and IgG_2_ in the colostrum and daily protein intake in the 100 days before birth. Additionally, different energy concentrations in the feed during the dry period (56 to 8 days before birth) did not influence the total Ig concentration, nor the concentration of IgG or IgM. However, the colostrum from cows in the “high energy” group had significantly (*p* < 0.01) higher concentrations of IgA compared to that from cows in the “low energy” group [110]. Similarly, Mann et al. (2016) [111] investigated the effect of different dry period feeding management practices on the IgG concentration in colostrum. Cows fed a restricted energy diet during the dry period showed a higher IgG concentration, whereas cows fed a higher energy density diet produced colostrum with lower IgG concentrations. As such, under some conditions, energy deficiency may impair the Ig concentration.

## 4. Conclusions

On dairy farms, calf rearing and its associated management processes are of particular importance since healthy calves are the basis for the (further) development of the farm. In addition, calf rearing is also receiving increasing public attention. Many studies have investigated the various aspects of colostrum management, the factors that influence Ig concentration, and Ig concentration measurement techniques. Studies have shown that colostrum management, in particular, is a decisive factor in calves’ health maintenance and survival, and thus forms the basis for their well-being. A high Ig concentration in the colostrum is a key component for successful colostrum management. This review has summarized, compared, and discussed the most important results in this research area. Thus, it contributes to a transparent presentation of significant findings and identifying the remaining problems in this context.

Different methods permit the estimation of Ig concentrations. Direct methods, such as RID and an ELISA, represent the gold standard. A TIA and IR spectroscopy are other laboratory methods. Nonetheless, the direct methods described in this review are not practical for use on farms; they are time consuming, and the results are not available within 3 h. Moreover, since these are laboratory methods, specific procedures must be followed, and their performance is not intuitive. Furthermore, these methods require special reagents that would have to be ordered. The application of the methods would also have to be shown to the farmers by trained personnel. In addition, initial supervision would be necessary to ensure proper execution and meaningful results—all in all, they are very time-consuming and labor-intensive methods. The user must have a high level of qualification for using these direct measurement methods.

Nevertheless, with respect to the significance of their results, direct measurement methods are better than indirect methods. It may be possible to develop practical variants of direct laboratory measurement methods for on-farm use. Currently, only indirect methods, such as measurements using a refractometer and a colostrometer can be used on farms. For both methods, results have shown high correlations compared to RID. The refractometer is easier to handle than direct methods, and even easier to use than the colostrometer; it is a quick and safe method to measure Ig concentrations. Deriving the Ig concentration from the colostrum’s color or weight has been used for years, but is the least accurate method, primarily because it is based solely on visual perception.

Setting a cut-off value (<50 mg mL^−1^) reduces the amount of information that is obtained. If the colostrum intake of the calf is below the targeted 10–12% of the calf’s body mass, higher IgG concentrations are desirable to avoid an FPT. However, if only the commonly used threshold of 50 mg mL^−1^ is applied, information on the actual concentration is missing. There is much potential for improvement in the indirect measurement methods. To date, it is impossible to automatically measure the Ig concentration of colostrum and transfer the results directly into, for example, herd management practices. These data could be used to evaluate individual milkings and identify the potential causes of diseases or long-term monitoring. Linking colostrum data with other health data of the calf or cow is also not feasible. As there is currently no possibility to store and process colostrum data automatically, its use in quality management has yet to be established. Digitally recording data would enable farmers to use it without much effort, thus optimizing their calf husbandry. The technical possibilities in this area have not yet been exhausted. For example, new methods already use QR codes and transmit the results to the farmer’s smartphone via an application.

The Ig concentration of colostrum is influenced by various factors, which can be categorized as animal- or environment-related factors. A high colostrum yield with a simultaneously low Ig concentration can lead to a strong dilution effect in the colostrum. For each additional liter of colostrum, there is a decrease in the Ig concentration. The number of lactations also influences the Ig concentration. The literature shows that the Ig concentration increases with the lactation number, particularly from the third lactation onward. Therefore, a division into primiparous and multiparous cows is not advantageous with respect to the Ig concentration.

In terms of influential factors, the number of lactations and breed should be considered when feeding colostrum, but no valuable colostrum should be discarded without control. Genetic effects in relation to the colostrum Ig concentration have only scarcely been studied but could play a role in the future. Furthermore, rapid milking and feeding after birth are essential, as the Ig concentration in the colostrum decreases and, at the same time, the calf’s absorption capacity for Ig declines. The first milking and feeding should take place within the first 2 h of life. To feed the calves colostrum containing high Ig concentrations as quickly as possible, even if the mother cow does not ensure such colostrum, frozen reserves of good colostrum are used to replace insufficient colostrum. Due to a gentle thawing process, the Ig concentration of previously frozen colostrum remains almost unchanged. Heating the frozen sample to 60 °C within 30 min and maintaining this temperature for another 120 min appears to be the safest option, as numerous studies have shown that this leads to the lowest loss of IgG; however, it does involve an increased time requirement, which can be reduced to 15 min when heating by microwave, although this leads to a 44% loss.

Colostrum management practices have developed considerably in recent years and are becoming an increased focus with respect to improving calf husbandry and health. This development will continue to progress in the coming years, and further developed methods and more detailed studies of the influencing factors will further optimize the opportunities for farmers’ colostrum management practices.

## Figures and Tables

**Table 1:** Mean quantity and ratio of the Ig subtypes in bovine colostrum.

Ig	IgG		IgM	IgA	Source
	IgG_1_	IgG_2_			
Quantity (mg mL^−1^)	47.60	2.90	4.20	3.90	[3]
	75.00	1.90	4.90	4.40	[12]
	34.96	6.00	4.32	1.66	[13]
Ratio (%)	85–95		≤7	≤5	[4]

**Table 2:** Relationships between RID and ELISA measurements of IgG concentration in colostrum across different studies.

Reported Parameter (*p*)	Significant	Colostrum	Source
r = 0.36 (= 0.01)	Yes	fresh bovine	[33]
r = 0.12 (= 0.42)	No	heated bovine	
Ρ = 0.20 (<0.0001)	No	frozen caprine	[34]
R^2^ = 0.83 (<0.001)	No	frozen bovine	[35]

r, P = correlation coefficient between IgG concentration measured by RID and ELISA; R^2^ = coefficient of determination

**Table 3:** Sensitivities (Se), specificities (Sp), and correlations for measurements of IgG concentration in different studies with digital and optical refractometers in comparison to the gold standard.

Refractometer	Standard	Se	Sp	Correlation	R^2^	Special Features	Source
Digital Optical	RID	0.93	0.80	0.73 *	0.53	fresh colostrum for Se and Sp frozen colostrum for correlation	[20] ^1^
		0.91	0.85	0.71 *	0.51		
Digital Optical	RID	0.79	0.69	n. d.	n. d.	Incubated in water baths to maintain the optimum temperature	[10] ^1^
		0.56	0.90				
Digital Optical	RID	0.82	0.81	0.60 *	n. d.	n. d.	[5] ^1^
		0.80	0.83	0.60 *			
Digital Optical	RID	0.74	0.80	0.72 *	n. d.	frozen colostrum	[11] ^1^
		0.73	0.80	0.71 *			
Digital Optical	RID	1.00	0.66	0.74	n. d.	frozen and unheated colostrum	[42] ^1^
		1.00	0.63	0.73			
Digital	RID	0.97	0.61	0.75		frozen and heated at 60 °C for 30 min	
Optical		0.97	0.65	0.73			
Digital Optical	RID	0.97	0.65	0.71		frozen and heated at 60 °C for 60 min	
		0.97	0.68	0.70			
Digital Optical	RID	0.90	0.38	0.48		frozen and heated at 63 °C for 30 min	
		0.90	0.38	0.50			
Digital Optical	RID	0.88	0.39	0.58		frozen and heated at 63 °C for 60 min	
		0.88	0.39	0.57			
Digital	RID	0.75	0.78	n. d.	0.41	fresh colostrum for refractometer frozen colostrum for the RID	[1] ^1^
Digital	RID	0.66	0.83	0.64	0.43	frozen colostrum	[7] ^1^
Digital	RID	1.00	0.65	n. d.	n. d.	fresh colostrum for refractometer frozen colostrum for the RID	[53] ^1^
Digital	RID	0.84	0.79	0.71 *	n. d.	frozen colostrum	[45] ^1^
Digital	RID	0.84	0.79	0.68–0.76	n. d.	frozen colostrum	[54] ^2^
Optical	RID	0.93	0.66	0.75 **	0.56	frozen colostrum	[8] ^1^
Optical	ELISA	0.86	0.85	n. d.	0.43	frozen colostrum for ELISA n. d. for refractometer	[31] ^1^

n. d. = no data; R^2^ = coefficient of determination; ^1^ Cut-off point of ≥50 mg IgG mL^−1^ for colostrum of good quality; ^2^ Cut-off point of ≥150 mg IgG mL^−1^ for colostrum of good quality; * *p* < 0.001; ** *p* < 0.01.

**Table 4:** Sensitivities (Se), specificities (Sp), and correlations for measurements of IgG concentration in different studies with the colostrometer compared to the gold standard.

Standard	Se	Sp	Correlation	Source
RID	n. d.	n. d.	0.43	[58] ^1^
RID	0.75	0.78	n. d.	[1] ^1^
RID	0.76	0.66	n. d.	[1] ^1^
RID	n. d.	n. d.	0.67	[59] ^1^
RID	n. d.	n. d.	0.79	[44] ^1^
RID	0.84	0.77	0.77	[7] ^1^
RID	0.73	0.72	n. d.	[10] ^1^
RID	0.71	0.61	n. d.	[10] ^1^
RID	0.69	0.81	0.57	[5] ^1^
RID	n. d.	n. d.	0.83	[34] ^1^
ELISA	0.93	0.69	n. d.	[31] ^1^
Refractometer	n. d.	n. d.	0.89	[60] ^1^
Refractometer	n. d.	n. d.	0.86	[44] ^1^

n. d. = no data. ^1^ Cut-off point of ≥50 mg IgG mL^−1^ for colostrum of good quality

**Table 5:** Different concentrations (mg mL^−1^) of Ig subtypes measured in three studies at different time points postpartum.

Time Interval Postpartum	IgG (mg mL^−1^)	IgG (mg mL^−1^)	IgG_1_ (mg mL^−1^)	IgG_2_ (mg mL^−1^)	IgA (mg mL^−1^)	IgM (mg mL^−1^)
<6 h	96.7	113.0	90.0	2.8	1.6	4.5
6–11 h	82.1	94.0 (6 h) 82.0 (10 h)	79.0	1.9	1.7	4.0
>11 h	84.1	76.0	65.0	1.8	0.9	2.3
Source	[68]	[96]	[97]			
